# Bilateral Native Kidney Papillary Renal Cell Carcinomas in a 11-Year-Old Renal Transplant Patient

**DOI:** 10.1055/s-0042-1759546

**Published:** 2022-12-04

**Authors:** Çiğdem Ulukaya Durakbaşa, Deniz Ugurlu, Sabriye Gulcin Bozbeyoglu, Sinem Aydoner, Hatice Seneldir, Mehmet Onur Candir, Cengiz Candan, Atilla Gemici

**Affiliations:** 1 Department of Pediatric Surgery, Istanbul Medeniyet University Faculty of Medicine, Istanbul, Turkey; 2 Department of Pediatric Surgery, Goztepe Prof Dr Suleyman Yalcin City Hospital, Istanbul, Turkey; 3 Department of Radiology, Goztepe Prof Dr Suleyman Yalcin City Hospital, Istanbul, Turkey; 4 Department of Pathology, Istanbul Medeniyet University Faculty of Medicine, Istanbul, Turkey; 5 Department of Pediatric Oncology, Goztepe Prof Dr Suleyman Yalcin City Hospital, Istanbul, Turkey; 6 Department of Pediatric Nephrology, Istanbul Medeniyet University Faculty of Medicine, Uskudar, Istanbul, Turkey; 7 Department of Pediatric Nephrology, Baskent University Istanbul Hospital, Istanbul, Turkey

**Keywords:** renal cell carcinoma, bilateral, transplantation, native kidney, child

## Abstract

Renal cell carcinomas (RCCs) are the most common renal tumors in adults and are usually sporadic and unilateral. Renal transplant recipients have an increased risk of developing RCC. RCC development after kidney transplantation is very rarely reported in children. We present a 11-year-old boy who had cadaveric kidney transplantation for kidney failure 2 years ago. He was under immunosuppressive therapy and presented with microscopic hematuria. An ultrasound (US) revealed bilateral solid renal masses. Further cross-sectional imaging showed a 60 × 70 × 60-mm right renal mass with claw sign and a 5 × 6 × 6-mm mass in the left renal lower pole. A bilateral radical nephroureterectomy of native kidneys was performed. The pathology revealed bilateral papillary RCC without TFE3 upregulation. The patient was kept on low-dose immunosuppressive therapy in the perioperative period. He received no chemotherapy but a close radiological surveillance was undertaken. He is tumor-free 2 years after the operation. RCC is a rare tumor for children and bilateralism is even rarer. The child had a history of chronic kidney disease, peritoneal dialysis, and immunosuppressive therapy. As there are no standardized protocols regarding imaging in transplanted kidneys routine surveillance, US follow-up should also focus on detecting malignancy.

## Introduction

The most common renal tumor in adults is renal cell carcinoma (RCC). 1 Most are sporadic and unilateral. 1 RCC is rarely encountered in childhood and accounts for only approximately 5% of all pediatric renal tumors. 2 3 Of all patients, 1 to 4% have bilateral tumors and bilateralism is more common in the hereditary forms of the disease. 4 RCC developing in children are generally indistinguishable by preoperative imaging from more commonly occurring primary pediatric renal tumors, especially Wilms' tumor. The ultimate diagnosis is possible by histological examination.

It is known that patients with end-stage renal disease on dialysis or after transplantation are at increased risk for cancer development. 1 Renal transplant patients of all age groups are at higher risk of developing skin cancers and lymphoproliferative disorders with immunosuppression as a known contributing factor. 5 The incidence of renal tumor development is also known to be increased after kidney transplantation. These tumors predominantly develop in native kidneys and are almost always unilateral. 6 7 Tumor development in the native kidneys after transplantation is very rarely reported in children. 7 We report a child aged 11 years without any genetic susceptibility who developed bilateral RCC 2 years after kidney transplantation.

## Case Report

**Fig. 1:**
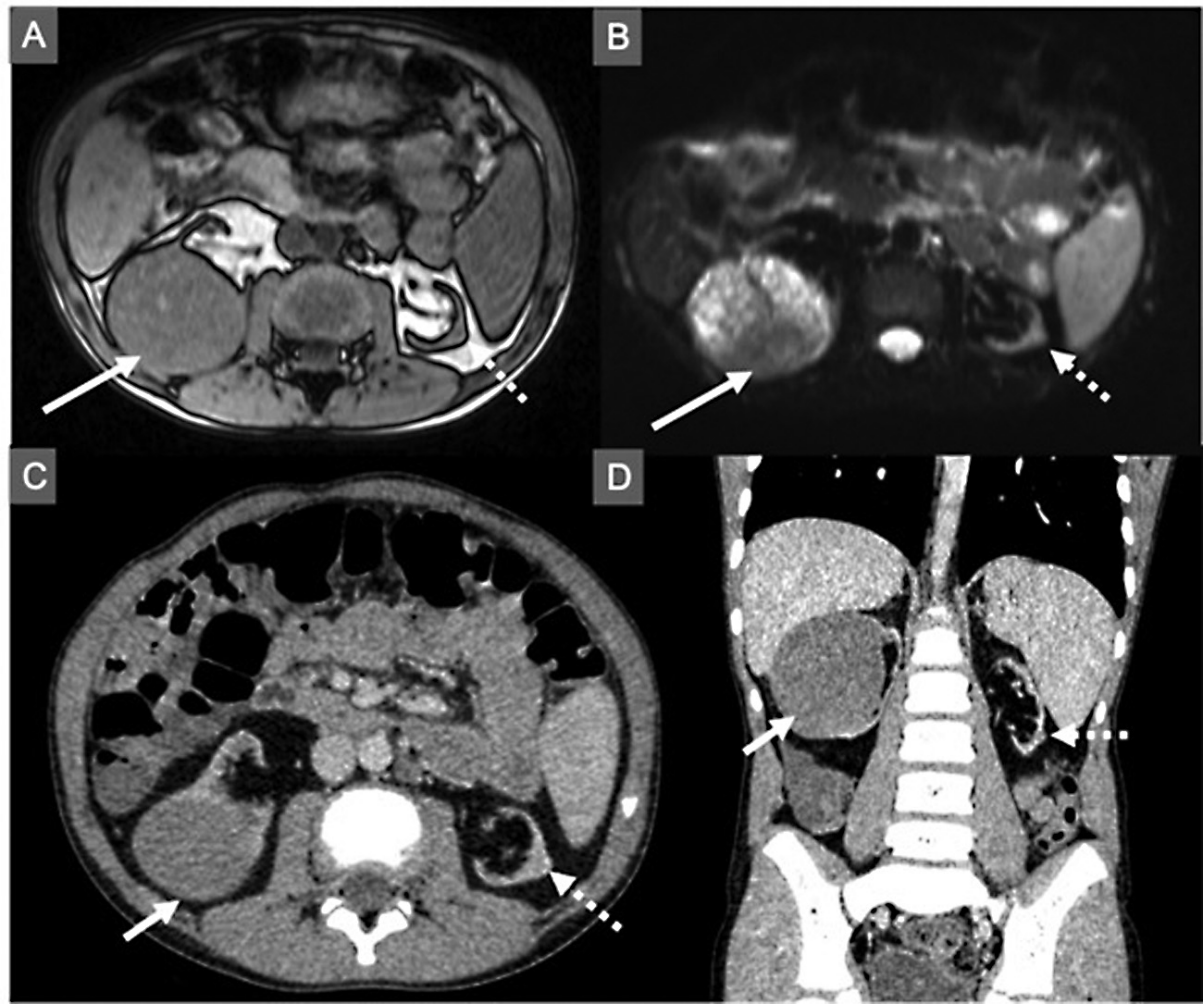
Cross-sectional imaging of the patient ( *arrow* : right renal mass; *broken arrow* : left renal mass). ( **A** ) Out-of-phase T1 magnetic resonance sequence shows absence of fat. ( **B** ) Diffusion-weighted T2 magnetic resonance sequence shows restricted diffusion. ( **C, D** ) Contrast enhanced axial and coronal computerized tomography images show contrast uptake by both lesions.

**Fig. 2:**
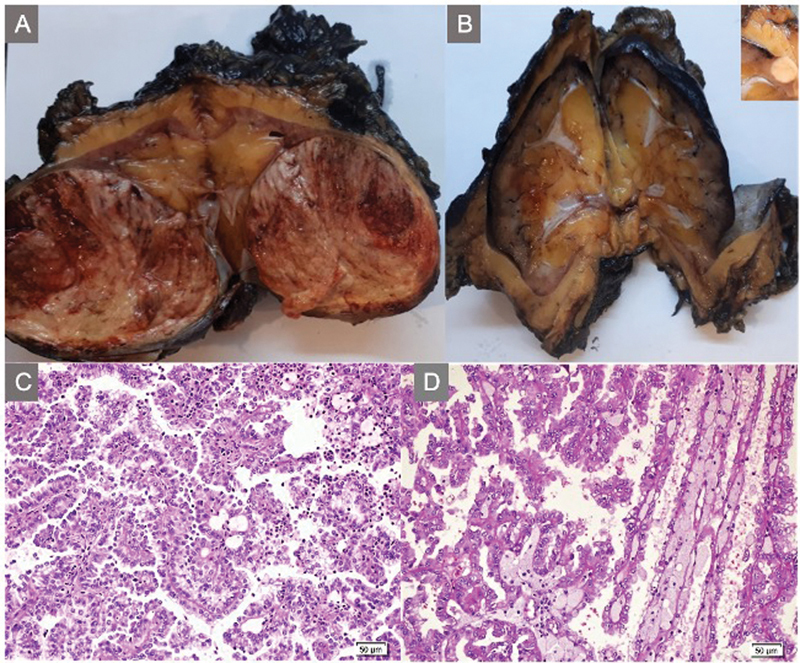
( **A** ) The excised right-sided tumor with prominent pseudocapsule which is friable and necrotic with areas of hemorrhage. ( **B** ) Sectioning of the atrophic left kidney revealed the mass in renal parenchyma detected in horizontal serial sections *(inset)* . ( **C** ) Microscopic examination revealed papillae or tubulopapillary architecture with fibrovascular cores. Papillae were lined by a single layer of cells with scant amount of pale cytoplasm and low nuclear grade (H&Ex200). ( **D** ) The papillae contained numerous foamy macrophages (H&Ex200).

**Fig. 3:**
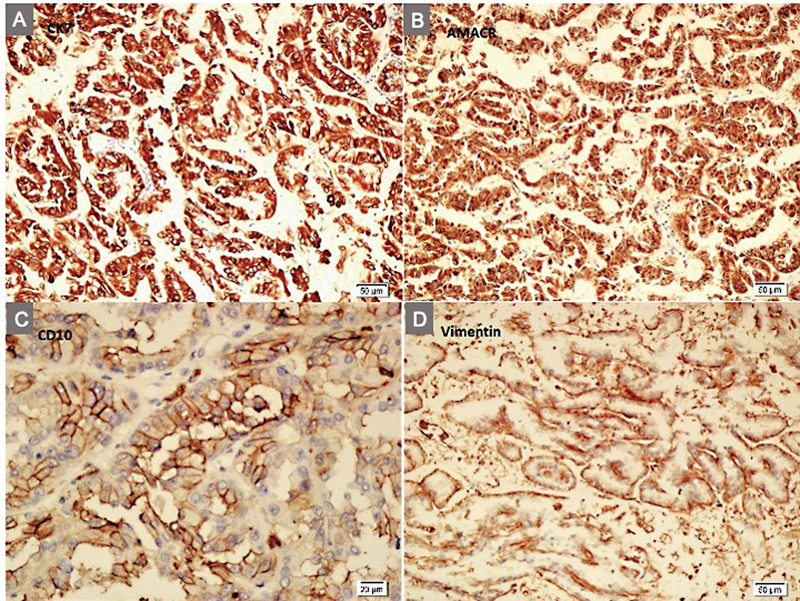
( **A–D** ) Tumoral cells expressed CK7 (x200), AMACR (x200), CD10 (x400), and vimentin (x200).

A 11-year-old boy presented with microscopic hematuria which was detected during a routine follow-up visit. He was diagnosed with focal segmental granulosis at the age of 2.5 years and received peritoneal dialysis (PD) for 4 years beginning at the age of 5 years. He had cadaveric kidney transplantation for kidney failure 2 years before the current presentation. The immunosuppressive therapy consisted of tacrolimus (serum level 6.1 ng/mL), mycophenolate sodium, and prednisolone. Evaluation in the outpatient clinics was done every 2 months as a part of regular follow-up protocol. He had missed a previous follow-up appointment because of the fear of the COVID-19 pandemic. An ultrasound (US) scan revealed, hypoechoic, heterogeneous solid mass measuring 6 × 7 × 6 cm in the right-sided native kidney and 5 × 6 × 6 mm isoechoic solid mass in the lower pole of the left native kidney. Doppler ultrasound showed both renal veins and vena cava were free of thrombus and patent. A non-contrast magnetic resonance imaging followed by computerized tomography with contrast showed a hypodense heterogeneous capsulated solid mass with 55 to 56 HU and showing claw sign without calcification in the right kidney. It was iso-/hypo-intense on T1 and hyperintense on T2 images ( Fig. 1 ). There was a left lower lobe heterogeneous mass which was iso-intense on T1 and iso-/hyper-intense on T2 images. The parenchymas of both native kidneys were thin and atrophic in accordance with a renal disorder. Because of presumptive diagnosis of a renal malignancy, mycophenolate sodium was stopped, tacrolimus dose was decreased (to be kept at 4 ng/mL serum level), and prednisolone dose was increased. He underwent a laparotomy with bilateral radical nephroureterectomy. The postoperative course was uneventful. The histopathological examination revealed a right-sided 70 × 60 × 50-mm and a left-sided 6 × 6 × 5-mm papillary RCCs both with CK7, AMACR, CD10, and vimentin expression ( Figs. 2 and 3 ). TFE3 or WT1 expressions were negative. Both tumors were limited to the kidneys without regional lymph node metastasis or lympho-vascular invasion. A postoperative FDG-PET scan showed no residual or metastatic lesions. Mycophenolate sodium was re-started, tacrolimus dose was increased to keep at a serum level of 5 mg/mL and prednisolone dose was decreased. The patient was scheduled for regular follow-up visits with ultrasound scanning every 3 months for the first year and every 6 months thereafter. He did not receive further treatment for RCC. A rejection was not encountered with his panel reactive antibody and donor-specific antibodies levels remaining negative. He is currently free of malignant disease 2 years after the operation with a current BUN level of 16 mg/dL and creatinine level of 0.66 mg/dL.

## Discussion

RCC constitutes 80 to 85% of all adult renal neoplasms and 5% of all pediatric renal neoplasms. 2 Most adults have clear-cell RCC type of the tumor and most children have translocation RCC followed by papillary morphology. 1 2 3 8 Translocation variant can be screened by TFE3 immunohistochemistry with a 100% sensitivity. 3 8 In our patient, the histopathology of the resected specimens was typical for papillary RCC with cells arranged in tubular and papillary configurations with abundant foamy macrophages and besides, TFE3 expression was negative. In general, RCC can be bilateral in 1 to 4% of all patients and bilateralism is more common in the hereditary forms of the disease than in the sporadic. 4 In a large pediatric RCC series which included 93 children <18 years of age, translocation type RCC accounted for 52% of the tumors and papillary for 13%. 3 Among these, two (2%) were bilateral at the time of diagnosis.

Posttransplant RCCs mostly develop in one of the native kidneys with a reported incidence of <1.5%. 7 9 The risk of developing RCC in renal transplant patients is reported to be 15 to 100 times higher than in the general population. 6 9 Although, a great majority are clear-cell RCC followed by papillary subtype, papillary RCC is more frequently observed than in non-transplanted patients. 6 The risk of developing RCC in pediatric transplant recipients is unknown. A study in North American pediatric renal transplant recipients showed that the most commonly observed post-transplant “solid tumor” was RCC with three cases out of a total of five developing in native kidneys in over 10,000 enrolled patients. 5 There has been only one previous pediatric report with bilateral RCC of native kidneys which was observed in a 16-year-old transplant recipient. 7 The diagnosis was papillary RCC and the ultimate outcome after surgery was rejection with chronic allograft nephropathy most likely due to discontinuation of immunosuppression.

The mechanisms leading to an increased risk for RCC development in transplant patients are not fully understood. Duration and type of immunosuppression, the primary native kidney disease, and duration of hemodialysis before transplantation are regarded as among risk factors in adults. 6 Although our patient was apparently receiving immunosuppressive therapy, one study found out immunosuppression did not increase the risk of developing native kidney malignancy, unlike other locations like the lymphoproliferative system. 9 The authors did not find a significant relationship between RCC development and immunosuppression and they postulated the underlying renal disorder causing renal failure and/or hemodialysis are contributing factors. Our patient did not receive hemodialysis but had undergone PD therapy before transplantation. The effect of PD on developing renal malignancy has not been studied in detail. In one study, the prevalence of RCC development in PD patients was found to be 1.3% and the authors suggested a regular follow-up protocol for cancer in PD patients. 10

COVID-19 pandemic has put a burden on health care systems all over the world. Among these are limited access to the health care and fear of increased infective risks especially when transplant and cancer patients are concerned. 11 Our patient missed a regular follow-up visit because of that fear with a resultant 4-month gap in evaluation before the tumors were diagnosed. Bilateral RCCs can develop synchronously or be metachronous. If the tumors are diagnosed concomitantly or within 3 months of the former tumor they are defined as synchronous bilateral RCCs. 12 With the available data, we cannot draw a definite conclusion whether the presented child had metachronous or synchronous tumors.

In children, the median age at RCC diagnosis is 11 years which is higher in respect to Wilms' tumor. 3 Our preoperative diagnosis was in favor of RCC because of the age of the presented patient, but Wilms' tumor could only be excluded after pathological examination. Under normal circumstances, bilateralism of a renal tumor is a challenge for the surgeon and partial nephrectomy is an integral part of the treatment plan. However, because the tumors were in the non-functioning native kidneys, a curative bilateral nephroureterectomy was a straightforward decision in our patient.

Malignant lesions can behave more aggressively in transplant recipients because of immunosuppression. The modification of the immunosuppressive treatment was a challenge in our case because the data about the alterations in the anti-rejection therapy for patients with newly developed renal malignancies is very limited. 1 9 We modified the treatment so that the immunosuppression as well as the probability of rejection are minimized.

Although kidney transplant recipients are at an increased risk for RCC development, the reported cumulative incidence is still low resulting in an inconsistency in the screening recommendations for such malignancies after transplantation. 1 6 Most transplant units do not have protocols for early detection of RCC development in native kidneys. Given the longer life expectancy of children, a focused screening program for pediatric transplant patients should be a future subject for pediatric transplant community.

## Conclusion

Unlike adults, RCC is a rarely diagnosed malignancy in children. Bilateral RCC developing after kidney transplantation in a child is even rarer with only one previous report. The presented child with bilateral papillary RCC had a history of chronic kidney disease, PD, and immunosuppressive therapy. The contribution of these factors to RCC development has not been clearly defined. Localized RCC is treated by radical nephroureterectomy which is regarded curative. Routine surveillance ultrasound follow-up for renal tumor development in children who underwent renal transplantation should be considered.

## Notes

New Insights and Importance for a Pediatric Surgeon Both renal cell carcinomas and bilateral involvement are rare in children. We report a renal transplant patient who was under immunosuppression and successfully treated with bilateral nephroureterectomy of the native kidneys.
